# Dispersion of the soybean root rot by *Cycloneda sanguinea* (Coleoptera: Coccinellidae)

**DOI:** 10.1038/s41598-018-20587-8

**Published:** 2018-02-05

**Authors:** Geraldo Salgado-Neto, Marcos André Braz Vaz, Jerson Vanderlei Carús Guedes, Marlove Fátima Brião Muniz, Elena Blume, Carlos Frederico Wilcken, Bárbara Monteiro de Castro e Castro, Angelica Plata-Rueda, José Cola Zanuncio

**Affiliations:** 1 Departamento de Defesa Fitossanitária, Universidade Federal de Santa Maria, Santa Maria, 97105-900 Brazil; 2 Departamento de Estatística, Universidade Federal de Santa Maria, Santa Maria, 97105-900 Brazil; 3 Departamento de Entomologia, Universidade Federal de Viçosa, Rio Paranaíba, 38810-000 Brazil; 4 Departamento de Proteção de Plantas, Escola de Ciências Agronômicas, Universidade Estadual Paulista, 18603-970 Botucatu, Brazil; 5 Departamento de Fitotecnia, Universidade Federal de Viçosa, 36570-900 Viçosa, Minas Gerais Brazil; 6 Departamento de Entomologia/BIOAGRO, Universidade Federal de Viçosa, Viçosa, 36570-900 Brazil

## Abstract

The dispersion of pathogenic microorganisms consists of the transport of pathogens from their source to inoculate a new host. Agricultural and economic importance of the Soybean root rot justifies studying this disease, especially the role of insects as dispersers. The aim of this study was to evaluate the role of the ladybird beetle, *Cycloneda sanguinea* Linnaeus (Coleoptera: Coccinellidae) in the dispersion of pathogens that cause Soybean root rot. Three pathogen species, *Macrophomina phaseolina* (Tassi) (Sphaeropsidales: Botryosphaeriaceae), *Fusarium incarnatum-equiseti* species complex (FIESC), and *F. commune* (Skovgaard) O’Donnell & Nirenberg were isolated from the midgut of ladybird beetles and cultured. *Macrophomina phaseolina* was identified by morphology while for the other two species, DNA was sequenced. The DNA extracted was amplified in the Internal Transcriber Spacer (ITS) region, sequenced and compared to voucher sequences deposited in the GenBank. Sequences of nucleotide ITS1-5.8 S were identified in the regions of rDNA-ITS4 ribosomal DNA. This is the first report of *Macrophomina phaseolina* (Tassi) (Sphaeropsidales: Botryosphaeriaceae), *Fusarium incarnatum-equiseti* species complex (FIESC), and *F. commune* (Skovgaard) O’Donnell & Nirenberg, being dispersed by *C. sanguinea* in Brazilian soybean fields.

## Introduction

Infectious disease has a major influence on the demography of human, plant, and animal populations. Plant pathogen dispersion is a process of movement of propagules from one host to a new host. In this context, plant pathogens produce a large number of propagules with most of them lost before reaching the host plant. Survival strategies of microorganisms such as hyphae and spores in an insect-vector enable the primary cycle initiation of a disease. Insects transmit some plant diseases and can retain the pathogens in the absence of host plants, contributing to the pathogen’s survival^1^. Some microorganisms can multiply, reproduce, and undergo cyclic changes inside an insect vector^1–5^.

*Fusarium* (Hypocreales: Nectriaceae) species cause rot diseases in different crops in South America. *Fusarium incarnatum* (Roberge) Saccardo was isolated from soil and aerial plant parts in banana and palm trees. This fungus has been implicated in plant diseases, including walnut canker, kangaroo paw blight of ornamental plants, bean pod and seed rot, reducing sorghum seed germination and seedling growth, melon corky dry rot and storage rot problems in mushrooms, besides being the dominant fungus in pearl millet grain^6^. *Fusarium equiseti* (Corda) Saccardo is a cosmopolitan soil inhabitant worldwide, mainly in dry areas. Damage caused by this fungus occurs in senescent plant tissue and produces diseases such as the sour cherry tree cankers, rot in pumpkin and cucurbit fruits in contact with soil, and diseases in the date palm^6^. *Fusarium commune* Skovgaard, O’Donnell & Nirenberg was isolated from soil and pea in Denmark and it is widespread in the Northern hemisphere in different crops such as carnation, corn, carrot, barley, and western white pine^7^. In tropical areas, *F. commune* was isolated from Dajiao bananas in Guangdong, China and Cavendish bananas in Brazil^8^. *Fusarium commune* caused damping-off and seedling root rot on soybean in the United States^9^.

Many different insect species are known to vector *Fusarium* species such as *Xylosandrus compactus* (Eichhoff) (Coleoptera: Curculionidae)^10^, *Dendroctonus* sp. (Coleoptera: Curculionidae)^11^, *Reticulitermes flavipes* (Kollar) (Blattodea: Rhinotermitidae)^12^, *Diabrotica speciosa* (Germar) (Coleoptera: Chrysomelidae)^13^, *Spodoptera littorallis* (Boisduval) (Lepidoptera: Noctuidae)^14^, *Xyleborus fornicatus* (Eichhorn) (Coleoptera: Curculionidae)^15^, *Triatoma* sp. (Hemiptera: Reduviidae)^16,17^, *Hypothenemus hampei* (Ferrari) (Coleoptera: Curculionidae)^16,18^, *Cyclocephala modesta* Burm, *Dyscinetus gagates* Burm, and *Diloboderus abderus* Sturm (Coleoptera: Scarabaeidae)^5^.

The ladybird beetle, *Cycloneda sanguinea* Linnaeus (Coleoptera: Coccinellidae) occurs in several countries in the Americas^19^. Larvae of *C. sanguinea* feed voraciously on aphids, ingesting fluid from their body, while adults devour the aphids completely^19,20^. This insect is a predator of *Aphis gossypii* Glover, *Aphis papaveris* Fabricius, *Hyadaphis foeniculi, Hyalopterus pruni* Geoffroy, *Macrosiphum euphorbiae* Thomas, *Macrosiphum rosae* Linnaeus, *Macrosiphum persicae* Sulzer, *Ropalosiphum maidis* Fitch, *Toxoptera aurantii* (Boyer de Fonscolombe) and *Schizaphis graminum* Rondani (Aphididae)^3,18–22^.

The increasing incidence of the soybean root rot in Southern Brazil justifies researches on insects dispersing and spreading inoculum of this disease. The aim of this study was to evaluate the dispersal of fungal root pathogens, by isolating the fungi of *C. sanguinea* collected in soybean fields, followed by a pathogenicity test in healthy soybean plants.

## Results

### Molecular fungi identification

**Figure 1:**
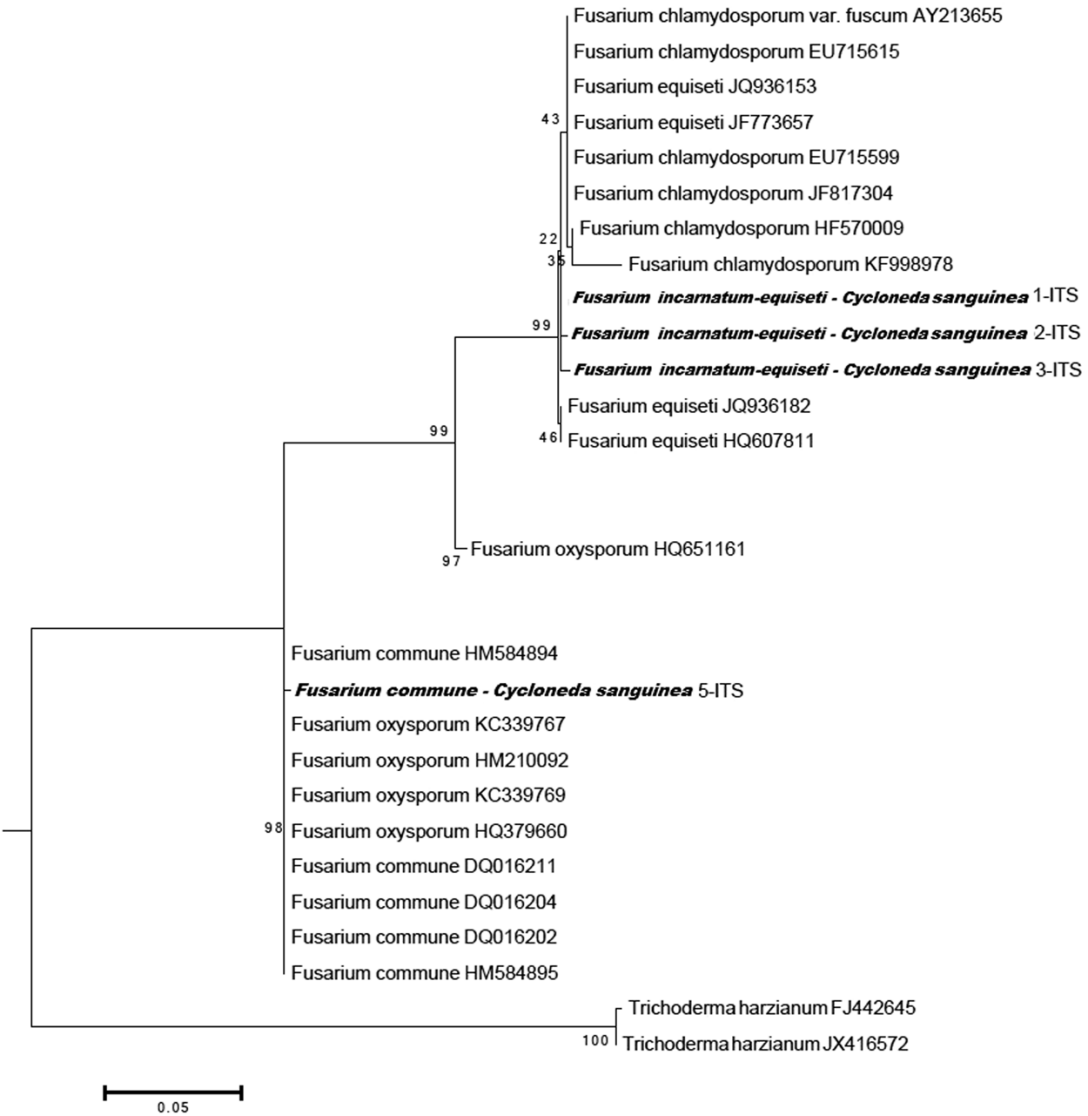
Phylogenetic dendrogram for the *Fusarium* isolates from the *Cycloneda sanguinea* midgut based in “Neighbor-joining” statistical method, derived from ITSrDNA regions. The evolutionary distances were calculated using the Tamura-Nei model. Number of branches represents the “bootstrap” number.

Three pathogen species were isolated from the midgut of *C. sanguinea*: *Macrophomina phaseolina*, *Fusarium incarnatum-equiseti* and *F. commune*. Fungi were identified based on morphological characteristics of *Macrophomina phaseolina* (Tassi) Goidanich. Specific identification of the other two species was possible by extracting and sequencing DNA from fungi isolated from the digestive tract of larvae and adults. Sequenced ITS1-5.8S-ITS4 regions of the ribosomal DNA were identified as *F. incarnatum-equiseti* complex species and *F. commune* (Fig. 1). GenBank accession numbers are KR082312 and KR082314.

### Association of detected pathogens with environmental parameters

The frequency of isolation of the fungal pathogens from the *C. sanguinea* was correlated with soil parameters and weather data. *Macrophomina phaseolina* had a positive relationship with Ca, Mg, Al, S, clay, P, K, Cu, Zn, pressure, temperature; and had a negative one with organic matter, pH (water), humidity and dew point. *Fusarium incarnatum-equiseti* and *F. commune* were negatively correlated with clay, P, Zn, humidity and rain; and a positive correlation with temperature and solar radiation per year (Table 1).

**Table 1:** Correlations between the frequency of isolation of *Fusarium* species and environmental parameters. Chemical analysis of the soil and weather data in Arroio Grande, Santa Maria, Rio Grande do Sul, Brazil. NS non-significant, *significant at 10% (P < 0.10), **significant at 5% (P < 0.05) by Pearson correlation analysis.

Parameters	*F. incarnatum-equiseti, F. commune*	*Macrophomina phaseolina*
**Elements**		
Calcium	−0.08 NS	0.49**
Magnesium	−0.04 NS	0.42**
Aluminium	0.02 NS	0.30**
Sulfur	0.15 NS	0.29**
Organic matter	0.16 NS	−0.28**
Clay	−0.20*	0.37**
Phosphorus	−0.22**	0.38**
Potassium	−0.13 NS	0.44**
Sulfur	−0.09 NS	0.39**
Zinc	−0.26**	0.53 **
Boron	0.05 NS	0.10 NS
Water pH	−0.01 NS	−0.39**
**Meteorological data**		
Temperature	0.41**	0.23**
Humidity	−0.22**	−0.42**
Dew point	0.01 NS	−0.50**
Pressure	0.05 NS	0.31 **
Wind speed	−0.12 NS	−0.14 NS
Radiation	0.30**	0.12 NS
Rain	−0.31**	−0.01 NS

### Pathogenicy tests

The healthy soybean plants inoculated with *F. incarnatum-equiseti* and *F. commune* isolates originating from the gastric cecum (digestive tract) of *C. sanguinea* larvae and adults showed the following symptoms of root rot: internerval leaf chlorosis, internerval necrosis and seedling damping-off and necrosis in shoots and branches. The *F. incarnatum-equiseti* and *F. commune* isolation frequency in soybean roots (12.50 ± 3.30% SD) also reduced the seed germination (average 87.50 ± 33.07% SD), lap diameter (average 0.51 ± 0.20% SD) during plant reproduction phase and, increased leaf (2.00 ± 0.89% SD) and root (1.75 ± 0.68% SD) symptoms. *Macrophomina phaseolina* was not tested for pathogenicity as it is an established pathogen of soybeans.

## Discussion

**Figure 2:**
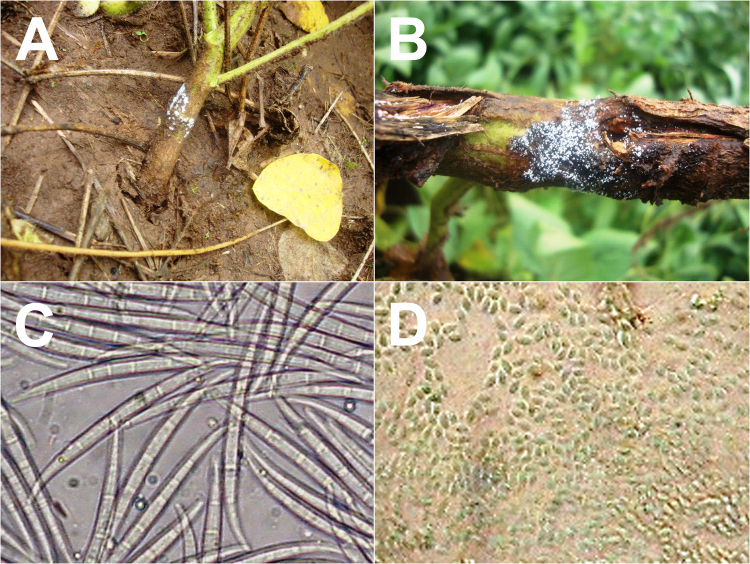
*Fusarium* species. (**A**) *Fusarium incarnatum-equiseti*. (**B**) *Fusarium commune* mass spores on soybean stem. (**C**) *Fusarium incarnatum-equiseti* macroconidia 3- to 5-septate curved spores (120 µm). (**D**) *Fusarium commune* microconidia aseptate oval or elliptical spores (4.2 µm). Figures (**A**–**D**) were obtained by Salgado-Neto.

*Fusarium* species isolated from the *C. sanguinea* digestive tract were morpho-physiologically identified and confirmed by molecular analysis of the ITS region of group 99, the *F. incarnatum-equiseti* species complex^23^. *Fusarium incarnatum* has a history of taxonomic and nomenclatural disagreements because of its isolate morphological variability^23^. *Fusarium equiseti* have two forms, macroconidia and microconidia. Macroconidia are strongly septate, sickle-shaped, and thick with a different curvature in the mycelium that is oval^6^. The sexual stage of *F. equiseti* is given by the specific epithet *Gibberella intricans* Wollenweber. *Fusarium incarnatum* also have macroconidia and microconidia, slender, with dorsal curved, and a straighter ventral surface (Fig. 2C). The apical cell is curved and tapers to a point; the basal cell is foot-shaped^24^. Microconidia are pyriform and uniseptate. The sexual or teleomorph stage of this fungus has not been described^6,24^.

**Figure 3:**
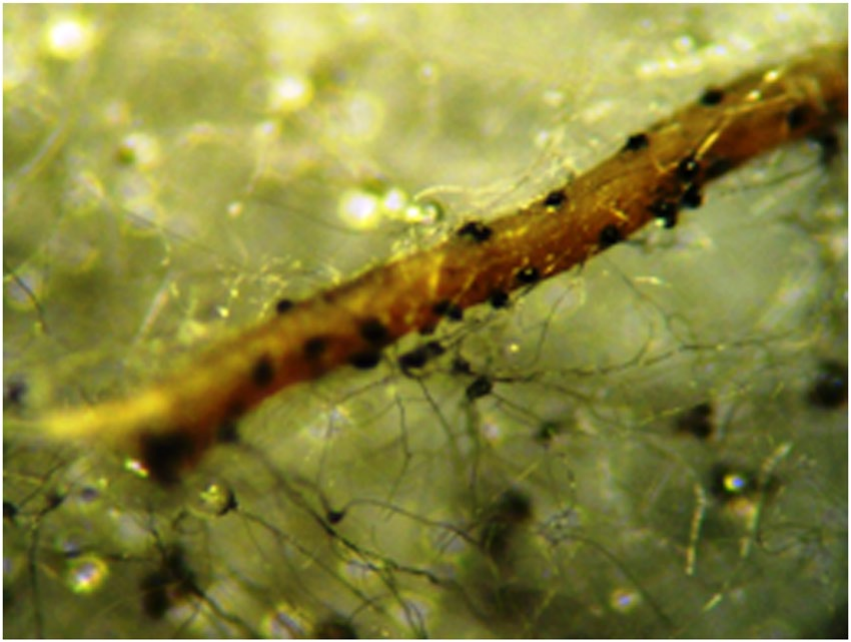
Pycnidial *Macrophomina phaseolina* structures (100–200 µm) of fungi on soybean roots. Figure was obtained by Salgado-Neto.

*Fusarium commune* have macroconidia and microconidia. Differing from other *Fusarium* species, the macroconidia of *F. commune* are short to medium long (3 to 5 µm), straight to slightly curved, slender, and thin-walled. The apical cell is tapered and curved, and sometimes with a sling hook^25^. Microconidia are oval, elliptical, or kidney shaped, usually aseptate (Fig. 3D). Conidia are produced on short monophialides in false heads, singly or in pairs on the aerial mycelium. The sexual or teleomorph stage of *F. commune* is unknown^7^.

*Macrophomina phaseolina* infect roots, stems, leaves, and fruits of various plant species in Brazil. It was first reported infecting beans and soybeans, causing charcoal rot disease with numerous tiny, black, and irregularly shaped microsclerotia (Fig. 3). For this fungus, these microsclerotia are survival structures allowing it to persist for a long time in the soil. Transport of contaminated soil during soil tillage and preparation is the one of the main dispersal methods of *M. phaseolina* within and between fields, and consequently, this fungus has become a globally important crop pathogen^26,27^. However, *M. phaseolina* lost its ability to reproduce sexually, or can do it only under special environmental conditions^26–28^.

The presence of *F. incarnatum-equiseti* and *F. commune* spores and hyphae was confirmed into midgut and mouthparts of *C. sanguinea* on soybean roots and stems. Similar studies have indicated a symbiotic relationship between *F. solani* and *H. hampei*^16^. Larvae of Scarabaiedae such as *C. modesta*, *D. gagates*, and *D. abderus* may spread *F. oxysporum* inoculum^5^. *Fusarium* spores are abundant in nature and can be transmitted and dispersed when passing ungerminated through insect alimentary tracts. Passage of *Fusarium* spores through the midgut of *Musca domestica* L. (Muscidae: Diptera) did not alter the spores in their physical appearance or ability to germinate^29^. One potential biochemical capability detected in *Fusarium* isolates that may benefit insect hosts include sterol synthesis, detoxification of (plant or venomous preys) derived allelochemicals and secondary metabolites, and nitrogen scavenging from unconventional substrates, including cyanide and formamide^30^. The presence of *Fusarium* species in the midgut suggests that *C. sanguinea* may be harboring this fungus in order to benefit from its metabolic capabilities.

Coccinellids are mycophagous, foraging in patches of asexual spores, may acquire conidial inoculum acting as mechanical vectors of pathogens^31,32^. The fungal spores are attractive as food for coccinellids, and therefore should not survive digestion. However, spores remained viable in the mycophagy Halyziini-powdery mildew system^31,32^. Another system involves a diet of aphids present on apple for the coccinellid *Hippodamia convergens* Guérin-Méneville carrying the fungal pathogen *Discula destructiva* Redlin (dogwood anthracnose) excreting viable spores^31–33^. In this context, spores *Fusarium* are found in plant tissues where phytophagous insects can acquire them via the cuticle or midgut, and, consequently, these spores are acquired by *C. sanguinea* through predation.

The presence of spores and hyphae of *F. incarnatum-equiseti* and *F. commune* in *C. sanguinea* indicates a close interaction between *C. sanguinea* larvae and the *Fusarium* complex. The teleomorph stage of *F. incarnatum* and *F. commune* is unknown but possibly occurs inside the midgut of larvae of *C. sanguinea*. The dispersion of *M. phaseolina*, *F. incarnatum-equiseti* and *F. commune* by *C. sanguinea* possibly starts with larval feeding (mycophagy, phytophagy, and zoophagy), absorbing spores and hyphae, near the soil in soybean stem as a protein and sugar source for this insect. The midgut of *C. sanguinea* can shelter pathogenic fungi so they may complete the reproductive cycle and disperse intact spores by excretion^34^.

High deficiencies or concentrations of N, P, K and micronutrients, and organic matter occurs in sandy and compacted soils, also linked to low pH reduces plant growth, favoring root rot development^35,36^. Mineral nutrients such as K, Ca, Mg, S, B, Mn and Fe favour healthy plants and avoid the entrance of external pathogens^37^. However, the effect of a nutrient on a particular combination (pathogen-host plant), cannot be generalized because some of them may increase disease severity, while others reduce it, as found here.

The weather factors affect the release and dispersal of *Fusarium* spores into the environment. A passively-released spore is a particle in a given area and its release from a pustule or conidiophore depends on mechanical force (caused by wind or rain) overcoming cohesive forces. Mechanical force from wind or rain is the main cause for particles adhering to plant surfaces to be removed. The chemicals exuded by splash-dispersed fungi, help spores to adhere to plant surfaces when they dry and also help the spores to be splashed when wet by reducing the surface tension of the water film. Changes in humidity, solar radiation and the atmospheric electric charge have been correlated with *Fusarium* spore mass release^38^. In this study, the mass release of *Fusarium* spores was observed after fog in the soybean crops (Fig. 2A,B).

This is the first study to report the presence *F. incarnatum-equiseti* species complex and *F. commune* with *Fusarium* disease symptoms dispersed by *C. sanguinea* in soybean crops. It is also the first report of *M. phaseolina*, which causes charcoal rot disease, and *F. incarnatum-equiseti* species complex, and *F. commune* simultaneously spread by *C. sanguinea*. This information contributes to our understanding of the geographical distribution of root phytopathogens in Brazil. Moreover, it brings new findings to the insect dispersing phytopathogens and to improve crop protection measures.

## Methods

### Local data and biological material

Experiments were carried out in the municipality of Santa Maria, Rio Grande do Sul state, Brazil (29.4056351°S, 53.4421171°W). Insect sampling was done four times a year on the seeds and roots of *G. max* between 2012 and 2013. Larvae and adults of *C. sanguinea* were collected in two soybean cultivars (BMX Power RR variety) with trenches (50 cm L × 25 cm W × 30 cm H) with 20 samples and 25 m distance.

### Molecular fungi identification

Larvae and adults of *C. sanguinea* were disinfected in ethanol and rinsed in sterile distilled water. A tube in which the beetle was immersed was covered by the barrier film, inverted three times, and sonicated for 2 min at 40 kHz (model 8851-34, Cole Parmer, Vernon Hills, IL); the rinse was repeated with sterile distilled water. The mouthparts, prothorax, cuticle, and midgut were dissected and placed in the Eppendorf tubes with 100 mL of 0.85% saline solution^18^ in the laboratory of phytopathology at the Universidade Federal de Santa Maria (UFSM). Then, samples of *C. sanguinea* in saline solution were added to Petri dishes with 3% PDA (Potato Dextrose Agar) media and incubated in a growth chamber (25 ± 6 °C, 75 ± 5% and a photoperiod 12:12 h [L:D]) for seven days^5^.

DNA was extracted in cetyltrimethylammonium bromide (CTAB)^39^. The mycelium was triturated in micro centrifuge tubes (1.5 mL, 10.8 × 40.6 mm) with the aid of a plastic pestle. The extracted genomic DNA was subjected to polymerase chain reaction (PCR) to amplify the ITS (Internal Transcribed Spacer) region located between the genes encoding the 18 S and 28 S ribosomal RNAs. The primers used were ITS1 (5′-TCCGTAGG TGAACCTGCGG-3′) and ITS4 (5′-TCCTCCGCTTATTGATATGC-3′).

Amplification and direct sequencing of fungi ribosomal RNA genes for phylogenetic analysis were conducted^40^. The products amplified were purified by precipitation with polyethylene glycol^41^, sequenced by the chain termination reaction method employing the reagent Big Dye 3.1 (Applied Biosystems), and analyzed in an automated capillary sequencer 3500 L (Applied Biosystems).

Species identification was based on the similarity of GenBank sequence databases. Similar sequences were searched in the GenBank using GenBank’s BLAST tool and aligned on the BioEdit program with the ClustalW algorithm. An outgroup was also chosen in the GenBank and aligned together to prepare a phylogenetic dendrogram using the MEGA5 software^42^.

A phylogenetic dendrogram was built for the *Fusarium* spp. isolates obtained from the midgut of *C. sanguinea* larvae and adults. Evolutionary distances were calculated and evolutionary history inferred with the Maximum Likelihood Method^43^. The branches show the percentage of trees with the associated taxa clustered together. Initial tree(s) for the heuristic search were obtained automatically. The maximum parsimony method was used when the number of common sites was <100 or lower than one-fourth of the total number of sites. The Bioinformatics Neighbor Joining (BIONJ) method with Maximum Composite Likelihood (MCL) distance matrix was used showing branches with the highest probability (log −1416.3605).

### Soil and weather analysis

In the soybean crops, soil was removed and sieved in a 2 mm metal wire mesh (0.8 × 0.4 m). The percentage of clay, organic matter, pH (water), and quantities of P, K, Ca, Mg, S, Al, Zn, B, and Cu from soil samples were analyzed at the “Centro de Ciências Rurais” of the “Universidade Federal de Santa Maria” (Santa Maria, Rio Grande do Sul, Brasil). Temperature (°C), humidity (%), dew point (%), pressure (hPa), wind speed (m/s), radiation (kJ/m²), and rainfall (mm) were obtained from the “Instituto Nacional de Meteorologia” (INMET) (Brazil) for the days that samples were taken.

### Pathogenicy test

Pathogenicity test was performed in plastic pots, (21 cm W × 17 cm H), filled with HA PLANTMAX substrate with two soybean seeds per pot. Pots were placed in a greenhouse under a natural photoperiod and temperature ranged from 25 to 30 °C during March, April, and May 2013. The three treatments, *Fusarium oxysporum* Schlechtendahl emend. Snyder and Hansen isolated from the alimentary the gut of *C. sanguinea* larvae (T1), *F. incarnatum-equiseti* and *F. commune* isolated from the gut of *C. sanguinea* larvae/adults (T2) and uninfected control (T3) were arranged in a completely randomized design with eight replications.

### Statistics

Data were analyzed by non-parametric Kruskal-Wallis test for stochaistic dominance. A Dunn’s test of multiple comparisons was also used for comparisons of the means at 5% significance level. Presence of *Fusarium* root and seed germination was analyzed using the exact binomial test (5% probability level) and compared with 2 × 2 treatments. The tests were performed with the R software^44^.
